# The hierarchy of evidence: Levels and grades of recommendation

**DOI:** 10.4103/0019-5413.30519

**Published:** 2007

**Authors:** BA Petrisor, M Bhandari

**Affiliations:** Division of Orthopedic Surgery, McMaster University, Hamilton, Ontario, Canada

Evidence-based medicine requires the integration of clinical judgment, recommendations from the best available evidence and the patient's values.^1^ The “best available evidence” is used quite frequently and in order to fully understand this one needs to have a clear knowledge of the hierarchy of evidence and how the integration of this evidence can be used to formulate a grade of recommendation.^2^ It is necessary to place the available literature into a hierarchy as this allows for a clearer communication when discussing studies, both in day-to-day activities such as teaching rounds or discussions with colleagues, but especially when conducting a systematic review so as to establish a recommendation for practice.^2^ This necessarily requires an understanding of both study design and quality as well as other aspects which can make placing the study within the hierarchy difficult.^3^ Another confounder is that there are a number of systems that can be used to place a study into a hierarchy and depending on the system a study can be placed at a different “level”.^4^ However, in general the different systems rate high quality evidence as “1” or “high” and low quality evidence as “4 or 5” or “low”. Recently, some orthopedic journals have adopted the reporting of levels of evidence with the individual studies and in many cases the grading system has been adopted from the Oxford centre for evidence-based medicine system.^3^ Rather than refer to any particular system we will speak in general terms of those studies deemed to be high-level evidence and relate this to those of lesser quality.

## LEVELS OF EVIDENCE

### Study design

Surgical literature can be broadly classified as those articles with a primary interest in therapy, prognosis, harm, economic analysis or those focusing on overviews to name a few.^5^ Within each classification there is a hierarchy of evidence, that is, some studies are better suited than others, to answer a question of therapy, for example, and may more accurately represent the “truth”. The ability of a study to do this rests on two main contributing factors, the study design and the study quality.^3^ In this context we will focus for the most part on those studies addressing therapy as this is generally the most common study in the orthopedic surgical literature.

Available therapeutic literature can be broadly categorized as those studies of an observational nature and those studies that have a randomized experimental design.^2^ The reason that studies are placed into a hierarchy is that those at the top are considered the “best evidence”.^5^ In the case of therapeutic trials this is the randomized controlled trial (RCT) and meta-analyses of RCTs. RCTs have within them, by the nature of randomization, an ability to help control bias.^6^,^7^ Bias (of which there are many types) can confound the outcome of a study such that the study may over or underestimate what the true treatment effect is.^8^ Randomization is able to achieve this by not only controlling for known prognostic variables but also and more importantly controlling for the unknown prognostic variables within a sample population.^7^ That is to say, the act of randomization should be able to create an equal distribution of prognostic variables (both known and unknown) within both the control and treatment groups within a study. This bias-controlling measure helps attain a more accurate estimation of the truth.^6^ Those studies of a more observational nature have within their designs areas of bias not present in the randomized trial.

Meta-analyses of randomized controlled trials in effect use the data from individual RCTs and statistically pool it.^5^,^9^ This effectively increases the number of patients that the data was obtained from, thereby increasing the effective sample size. The major drawback to this pooling is that it is dependent on the quality of RCTs that were used.^9^ For example, if three RCTs are in favor of a treatment and two are not or if the results show wide variation between the estimates of treatment effect with large confidence intervals (i.e. the precision of the point estimate of the treatment effect is poor) between different RCTs then there is some variable (or variables) causing inconsistent results between studies (one variable may in fact be differences in study quality among others) and the quality of usable results from statistical pooling will be poor. However, if five methodologically well done RCTs are used, all of which favor a treatment and have precise measures of treatment effect (i.e., narrow confidence intervals) then the data obtained from statistical pooling is much more believable. The former studies can be termed heterogeneous and the latter homogeneous.^9^

In contrast to this, the lowest level on the hierarchy (aside from expert opinion) is the case report and case series.^3^ These are usually retrospective in nature and have no comparison group. They are able to provide outcomes for only one subgroup of the population (those with the intervention). There is the potential for the introduction of bias especially if there is incomplete data collection or follow-up which may happen with retrospective study designs. Also, these studies are usually based on a single surgeon's or center's experience which may raise doubts as to the generalizability of the results. Even with these drawbacks, this study design may be useful in many ways. They can be used effectively for hypothesis generation as well as potentially providing information on rare disease entities or complications that may be associated with certain procedures or implants. For example, reporting of infection rates following a large series of tibial fractures treated with a reamed intramedullary nail^10^ or the rate of hardware failure of a particular implant to name a few.

The next level of study is the case-control. The case-control starts with a group who has had an outcome of interest and looks back at other similar individuals to see what factors may have been present in the study group and may be associated with the outcome. Let us take a hypothetical example. Those patients who have a nonunion following a tibial shaft fracture treated with an intramedullary nail. If one wanted to see what prognostic factors may have contributed to this, a group that was matched for the known prognostic variables such as age, treatment type, fracture pattern etc. could then be compared and an analysis of other prognostic variables such as smoking, nonsteroidal anti-inflammatory use or fracture pattern could be done to see if there was any association between these and the development of nonunion. The drawback to this design is that there may be unknown or as yet unidentified risk factors that would not be able to be analyzed. However, in those that are known, the strength of association may be determined and given in the form of odds ratios or sometimes relative risks. Other strengths of this study design are that they are usually less expensive to implement and can allow for a quicker “answer” to a specific question. They also can allow for analysis of multiple prognostic factors and relationships within these factors to help determine potential associations to the outcome of choice (in this case nonunion).

In contrast to the case-control and slightly higher on the levels of evidence hierarchy,^3^ the cohort study is usually done in a prospective fashion (although it can be done retrospectively) and usually follows two groups of patients. One of these groups has a risk factor or prognostic factor of interest and the other does not. The groups are followed to see what the rate of development of a disease or specific outcome is in those with the risk factor as compared to those without. Given that this is usually done prospectively it falls higher within the hierarchy as data collection and follow-up can be more closely monitored and attempts can be made to make them as complete and accurate as possible. This type of study design can be very powerful in some instances. For example, if one wanted to see what the effect of smoking was on nonunion rates, it wouldn't be ethical or generally possible to randomize patients with fractures into those who are going to smoke and those who are not. However, by following two groups of patients, smokers and non-smokers with tibial fractures for instance, one can then document nonunion rates between the two groups. In this case, because of its prospective design, groups can at least be matched to try and limit the bias of at least those prognostic variables that are known, such as age, fracture pattern or treatment type to name a few.

It is important to understand distinctions between study designs. Some investigators argue that well-constructed observational studies lead to similar conclusions as RCTs.^11^ However, others suggest that observational studies have a more significant potential to over or underestimate treatment effects. Indeed, examples are present in both medical and orthopedic surgical specialties showing that discrepant results can be found between randomized and nonrandomized trials.^6^,^8^,^12^ One recent nonsurgical example of this is hormone replacement therapy in postmenopausal women.^13^,^14^ Previous observational studies suggested that there was a significant effect of hormone replacement therapy on bone density with a favorable risk profile. However, a recent large RCT found an increasing incidence of detrimental cardiac and other adverse events in those undergoing hormone replacement therapy, risks which had heretofore been underestimated by observational studies.^13^,^14^ As a result of this the management of postmenopausal osteoporosis has undergone a shift in first-line therapy.^13^ In the orthopedic literature it has been suggested that when assessing randomized and nonrandomized trials using studies of arthroplasty vs. internal fixation, nonrandomized studies overestimated the risk of mortality following arthroplasty and underestimated the risk of revision surgery with arthroplasty.^8^ Interestingly, they also found that in those nonrandomized studies that had similar results to randomized studies, patient age, gender and fracture displacement were controlled for between groups.^8^ This illustrates the importance of both controlling for variables and for randomization which will control for potentially important but as yet unknown variables.

Thus the type of study design used places the study broadly into a hierarchy of evidence from the case series up to the randomized controlled trial. There is also, however, an internal hierarchy within the overall levels of evidence and that is usually based on the study methodology and overall quality.

## STUDY QUALITY

Concepts of study methodology are important to consider when placing a study into the levels of evidence. There are some that advocate dividing the hierarchy levels into sub-levels based in part on study methodology, while others suggest that poor methodology will take a study down a level.^2^,^3^ For instance, one RCT could be considered a very high-level study while another RCT because of methodological limitations may be considered lower. Do these then fall into separate categories or into sub-categories of the same level? It depends on the level of evidence system being used. The real point is that these systems acknowledge a difference in the quality and thus the “level” of these studies. In many instances however, the methodological limitations that will take a study down a level are not clearly defined and it is left to the individual to attempt to correctly categorize the study based on them. The rigor with which a study is conducted plays a role in how believable the results may be.^15^,^16^ Not all case-control, cohort or randomized studies are done to the same standards and thus if done multiple times, may have different results, both due to chance or due to confounding variables and biases. Briefly, if we take the example of a RCT one needs to look at all aspects of the methodology to see how rigorously the study was conducted. We present three examples of how different aspects of methodology may affect the results of a trial. While it is important to look closely at the methods section of a paper to see how the study was conducted, it must be remembered that if something has not been reported as being done (such as the method of randomization) it does not necessarily mean it was not done.^17^ This illustrates the importance of tools such as the “Consolidated Standards of Reporting” (CONSORT) statement for reporting trials which attempts to improve the quality of reporting.^18^,^19^

### Randomization

As randomization is the key to balancing prognostic variables, it is first necessary to determine how it was done. The most important concepts of randomization are that allocation is concealed and that the allocation is truly random. If it is known to which group a patient will be randomized it may be possible to potentially influence their allocation. Examples of this would include randomizing by chart number, birthdates or odd or even days. This necessarily introduces a selection bias which negates the effect of randomization. This makes concealment of allocation a vital component of successful randomization. Allocation can be concealed by having offsite randomization centers, web-based or phone-based randomization.

### Blinding

In surgical trials blinding is obviously not possible for some aspects of the trial. It is not possible (or ethical) to blind a surgeon, nor is it usually possible to blind a patient to a particular treatment. However, there are other aspects of a trial where blinding can play a role. For instance, it is possible to blind outcome assessors, the data analysts and potentially other outcomes' adjudicators. Thus it is important to understand who is doing the data collecting and ask, are they independent and were they blinded to the treatment received? If not, possible influences (either subconscious or not) on the patient and subsequent results can happen.

### Follow-up

The number lost to follow-up is very important to know as clearly this can affect the estimate of treatment effect. While some argue that only a 0% loss to follow-up fully ensures benefits of randomization,^20^ in general, the validity of a study may be threatened if more than 20% of patients are lost to follow-up.^5^ Calculations of results should include a worst case scenario, that is, those that are lost to follow-up are considered to have the worst outcome in the treatment group and those lost to follow-up in the control group having the best outcome. If there is still a treatment effect seen between the groups then this makes a more compelling argument for the treatment effect observed being a valid estimate of the truth.^21^

Scales have been devised that can rate a study based on its methodology and assign a score.^22^ This does not always need to be done in daily practice however. Knowledge of the different areas of methodology though may affect interpretation of the results and allow for the recognition of a “strong” study which may then provide more compelling and “believable” results as compared to a “weaker” study.

### Grades of recommendation

When truly does assessing the quality of a study in relation to the levels of evidence matter? It matters when a grade of recommendation is being developed. A very important concept is that a single high-level therapeutic study (in our case) does not imply a high grade of recommendation for treatment. A grade of recommendation can only be developed after a thorough systematic review of the literature and in many cases discussions with content experts.^2^,^4^,^23^ When developing grades of recommendation, it becomes important to place weights on studies with more weight being given to studies of high quality and high on the hierarchy and less so to lesser quality studies.^2^

The GRADE working group suggests a system for grading the quality of evidence obtained from a thorough systematic review [Table 1]. This should be done for all the outcomes of interest as well as all the potential harms. They suggest that once the total evidence has been graded then recommendations for treatment can be made.

**Table 1:** Criteria for assigning grade of evidence^22^

Type of evidence
Randomized trial = high quality
Quasi-randomized = moderate quality
Observational study = low quality
Any other evidence = very low quality
Decrease grade(s) if:
Serious (−1) or very serious (−2) limitation to study quality
Important inconsistency (−1)
Some (−1) or major (−2) uncertainty about directness
Imprecise or sparse data (−1)
High probability of reporting bias (−1)
Increase grade if:
Strong evidence of association - significant relative risk of >2 (<0.5) based on consistent evidence from two or more observational studies, with no plausible confounders (+1) Very strong evidence of association - significant relative risk of >5 (<0.2) based on direct evidence with no major threats to validity (+2)
Evidence of a dose response gradient (+1)
All plausible confounders would have reduced the effect (+1)

The GRADE working group suggests that when making a recommendation for treatment four areas should be considered: 1) What are the benefits vs. harms? Are there clear benefits to an intervention or are there more harms than good? 2) The quality of the evidence, 3) Are there modifying factors affecting the clinical setting such as the proximity of qualified persons able to carry out the intervention? and 4) What is the baseline risk for the potential population being treated?^1^ Once these factors are taken into consideration, the GRADE working group recommends a recommendation be placed into one of four categories. Either “do it” or “don't do it” and “probably do it” or “probably don't do it”. The grades of “do it” or “don't do it” are defined as “a judgment that most well-informed people would make”. The grades of “probably do it” or “probably don't do it” are defined as “a judgment that the majority of well-informed people would make but a substantial minority would not”.^2^

Thus one can see that a grade of recommendation in contradistinction to a level of recommendation is made based on the above four criteria. Inherent in the above criteria are a thorough review of the literature and a grading of the studies through knowledge of study design and methodology. Evidence-based medicine is touted as being a decision-making based on the composite of the triumvirate of clinical experience, the best available evidence and patient values. One can see that knowledge of the levels of evidence, the pros and cons of different study designs and how study methodology can affect the placement of a study within the hierarchy encompasses one aspect of this. The development of grades of recommendation based on the GRADE working group system gives one the tools to convey the best available evidence to the patient as well as help the literature guide the busy clinician. Also, different harms and benefits of various treatments are given different value judgments by individual patients. Discussions with patients about what is important to them, mixed with surgical experience and “what works in my hands” helps round out the decision-making when developing a treatment plan.
